# Evaluating Aesthetic Experience through Personal-Appearance Styles: A Behavioral and Electrophysiological Study

**DOI:** 10.1371/journal.pone.0115112

**Published:** 2014-12-31

**Authors:** Mei-chun Cheung, Derry Law, Joanne Yip

**Affiliations:** 1 Department of Social Work, The Chinese University of Hong Kong, Shatin, New Territories, Hong Kong SAR; 2 Institute of Textiles and Clothing, The Hong Kong Polytechnic University, Hung Hom, Kowloon, Hong Kong SAR; University of Groningen, Netherlands

## Abstract

Consumers' aesthetic experience has often been linked with the concept of beauty, which is regarded as subjective and may vary between individuals, cultures and places, and across time. With the advent of brain-imaging techniques, there is more and more evidence to suggest that aesthetic experience lies not only in the eye of the beholder, but also in the brain of the beholder. However, there are gaps in the previous research in this area, as several significant issues have not yet been addressed. Specifically, it is unclear whether the human brain really pays more attention and generates more positive emotional responses to beautiful things. To explore the brain activity relating to consumers' aesthetic experiences, 15 participants were recruited voluntarily to view a series of personal-appearance styles. They were invited to make aesthetic judgments while their brain activity was recorded by electroencephalography. Two electroencephalographic (EEG) indicators, theta coherence and frontal alpha symmetry, were utilized. Theta coherence is a measure of linear synchronization between signals at two electrode sites. It reflects the degree of functional cooperation between the underlying neuronal substrates and was used to explore the attentional processing involved in aesthetic judgments. Frontal alpha asymmetry is derived by subtracting the log-transformed absolute alpha power of the left hemisphere from the analogous log-transformed alpha power of the right hemisphere. It was used as an indicator of emotional response. During aesthetic judgments, long-range theta coherence increased in both hemispheres and more positive frontal alpha asymmetry was found when the styles were judged to be beautiful. Therefore, participants demonstrated brain activity suggestive of central executive processing and more positive emotional responses when they considered styles to be beautiful. The study provides some insight into the brain activity associated with consumers' aesthetic experiences, and suggests new directions for exploring consumer behavior from the perspective of neuroscience.

## Introduction

The interaction between personal preferences and social and cultural influences within a specific environmental context determines whether a piece of art, an object or a person is considered to be beautiful. Therefore, the perception or judgment of beauty is often regarded as subjective, and may vary between individuals, cultures and places, and across time. Leder et al. [1] proposed a psychological model of aesthetic appreciation to explicate the cognitive processes experienced when viewing an artwork. The model consists of five processing stages and two distinct outputs. The processing stages comprise perceptual analysis, implicit memory integration, explicit classification, cognitive mastering and evaluation. The outputs are aesthetic judgments and aesthetic emotion. Within this model, “aesthetic appreciation” refers to the cognitive processes involved in continuously upgrading affective states, which result in aesthetic emotion. Aesthetic judgment is the result of cognitive evaluation, and aesthetic emotion is a by-product of the processing stages. One merit of this model is its provision of an information-processing approach to the analysis of aesthetic appreciation, with feedback loops at certain stages. It also accommodates the influence of personal characteristics and social factors, along with the interaction between individual and environment. However, as this model is conceptualized from a general psychological perspective, and thus emphasizes the psychologically relevant features of artworks, it does not explicitly provide an explanation of how the brain engages in aesthetic appreciation. Therefore, it is difficult to investigate and identify the specific brain activity associated with each processing stage.

To gain a better understanding of the neural processes involved in aesthetic appreciation, Chatterjee proposed a general framework for the neural underpinnings of visual aesthetics from the perspective of visual cognitive neuroscience [2]. According to this model, the basic features of an artwork, like any other kinds of visual stimuli, are visually processed in the occipital brain regions. Attentional processing in the fronto-parietal circuits continuously modulates processing within the ventral visual system [3], [4]. The final stage involves decision making and the production of an emotional response, similar to the fifth processing stage of the model developed by Leder et al. [1]. Decision making is largely associated with activity in the dorsolateral frontal cortex, whereas the anterior medial temporal lobe, the medial orbitofrontal cortex and the subcortical region appear to be involved in the production of aesthetic emotion [2]. This neuropsychological model clearly identifies the brain regions involved in aesthetic appreciation, with evidence from visual cognitive neuroscience and neuropsychological studies of individuals with brain damage. It also provides an empirical neural framework that allows researchers to test hypotheses about the cognitive processing involved in aesthetic appreciation by conducting neuroimaging studies.

During the last few decades, brain-imaging techniques have provided numerous insights into the neural processing of cognitive functions by enabling researchers to identify the regions of the brain in which information is processed. The first stage of Chatterjee's model of aesthetic appreciation involves visual processing [2], and has been extensively explored in neuroimaging studies using functional magnetic resonance imaging (fMRI). Zeki and his research team identified certain visual cortical patterns associated with the processing of the crucial components of art [5] and proposed a theory of multistage integration in the visual brain [6]–[8]. For instance, the V4-complex is the “color center” in the brain [7], [9], [10]. Much attention has also been paid to the part of Chatterjee's model that involves decision making or aesthetic judgments [2]. Event-related potential studies have led to the development of a two-stage model [11]–[13]. During the first stage, which is associated with frontomedian activity, an initial impression is formed at around 300 ms after the presentation of a stimulus. The second stage is related to deep aesthetic judgments, and is associated with frontomedian and right-hemisphere activities, which occur at around 600 ms after the presentation of the stimulus [13]. Magnetoencephalography and fMRI studies have identified several brain regions associated with the aesthetic experience of beauty, such as the left dorsolateral prefrontal cortex [14], the medial orbitofrontal cortex (mOFC) [15], [16], and the left cingulate gyrus [17]. In a recent paper by Ishizu and Zeki [16], functional activity in a single region (field A1) of the mOFC was found to be associated with visual and musical beauty, and a linear association was identified between the strength of the blood oxygen level dependent signal and the subjective intensity of the experience of beauty. Ishizu and Zeki's findings have contributed to the formulation of a brain-based theory of beauty [16].

Consumers' aesthetic experience has often been linked with the concept of beauty, which is also regarded as subjective. The aesthetic significance of a fashion object, unlike that of other art forms, depends on how it appears on the human body. Therefore, some fashion objects “feel right” or “feel beautiful” when worn, whereas others “feel wrong” or “feel ugly” and are never worn. However, it is unclear whether our brain exhibits consistent neural patterns in response to fashion objects that “feel right” or “feel wrong.” During the last few decades, brain-imaging techniques have provided numerous insights into the neural correlates of beautiful faces [18], [19] and the neural basis of viewers' aesthetic appreciation of paintings [14]–[16]. To the best of our knowledge, however, few insights have been gained into consumers' aesthetic experience of personal-appearance styles from the perspective of cognitive neuroscience. The aim of this study was to investigate the neural processing involved in consumers' evaluation of personal-appearance styles to determine whether the human brain pays more attention and produces more positive emotional responses to personal-appearance styles that are regarded as beautiful. The findings have significant implications for fashion design and styling, and shed some light on the neuroscientific basis of fashion consumer behavior.

Chatterjee hypothesized that aesthetic appreciation is associated with attentional processing that involves the fronto-parietal circuits [2]. However, our current understanding of these circuits largely relies on neuroimaging studies of attention and neuropsychological research involving patients with brain lesions [4]. The fronto-parietal circuits have rarely been addressed in the context of aesthetic appreciation. Many neurophysiological studies have shown that both localized theta coherence and interregional theta coherence are associated with attentional processing. Specifically, localized theta activity represents the neural correlates of an attention system that allocate cognitive resources, whereas long-range frontal and posterior theta coherence indices are suggestive of memory-related executive functions [20]–[23]. Therefore, it is hypothesized that in comparison with a resting state in which individuals' eyes are open, the aesthetic judgments of appearance styles will elicit elevated theta coherence within and across the hemispheres of the brain.

According to Chatterjee's model, aesthetic emotion is an output of aesthetic appreciation [2]. Although researchers have identified links between certain cortical regions and the emotional processes involved in aesthetic appreciation [15], [17], [24], aesthetic emotion during aesthetic appreciation has rarely been measured directly. As emotion plays a large role in aesthetic appreciation, which is often regarded as a pleasurable experience, it is necessary to objectively identify aesthetic emotion in the brain of the perceiver. The results of numerous electroencephalographic (EEG) studies of affective processes have suggested that different emotions are associated with different EEG patterns in the frontal regions of the brain [25], [26]. Specifically, activation asymmetries in the anterior regions have been found to correlate with affective states. Positive emotions such as happiness are associated with greater left-sided activation [27]–[30], and negative emotions such as disgust are accompanied by greater right-sided activation [31], [32]. As alpha power is inversely associated with brain activation in the frontal region [33], [34], a positive asymmetry score denoting greater alpha power on the right and less alpha power on the left suggests greater left-sided activation; that is, a more positive emotional response. In contrast, a negative score represents greater activation on the right side, suggesting a more negative emotional response. Our empirical and clinical studies [35]–[37] have shown that frontal alpha asymmetry provides an effective and reliable means of distinguishing between positive and negative emotions. On the assumption that continuous success in cognitive mastering leads to positive changes in individuals' affective states [1], it is hypothesized that a positive value in the frontal alpha asymmetry index will be obtained if an individual judges an appearance style as beautiful, and a negative value will be obtained if an appearance style is perceived as ugly.

## Materials and Methods

### Participants

Fifteen university students (age: 21.13±1.41; years of education: 15.00; males: 3) from the Institute of Textiles and Clothing at The Hong Kong Polytechnic University were recruited to the study. They reported a negative history of neurological and psychiatric problems. The study was conducted in accordance with the Helsinki Declaration of the World Medical Association Assembly, and the research protocol was approved by the Human Subjects Ethics Sub-committee (HSESC) of The Hong Kong Polytechnic University. All of the students participated voluntarily, and were required to sign informed-consent forms prior to the study, in accordance with institutional guidelines.

### Stimuli Selection

The stimuli were collected by one of the co-authors (Law) for a qualitative paper exploring aesthetic preferences in the Chinese community [38]. To select the stimuli that represented personal-appearance styles being the most beautiful and the most ugly as perceived by participants, the whole collection of stimuli was first categorized into four major groups, namely “male fashion-oriented,” “male casual,” “female fashion-oriented” and “female casual.” Next, the stimuli were printed on sheets of A4 paper and given to 10 university students, who were invited to assess them as beautiful or ugly. In each of the 4 categories, the 10 stimuli assessed by the largest number of participants (7 or more) to represent personal-appearance styles being beautiful, along with the 10 stimuli judged by 7 or more of participants to represent personal-appearance styles being ugly, were selected. This selection process resulted in 40 personal-appearance styles perceived as beautiful and 40 personal-appearance styles perceived as ugly, which were subsequently used for data collection in the EEG experiment.

### EEG Recording

The EEG recordings were made using 64 Ag/AgCl-sintered electrodes mounted in a stretch-lycra Quik-Cap (Neuroscan, El Paso, TX, USA), with electrode placement in accordance with the international 10–10 system [39]–[41]. A ground electrode was placed on the forehead of each participant, anterior to Fz. The linked-ears reference scheme was used throughout the acquisition of data. Measurements of vertical electrooculography were taken between electrodes placed on the supraorbital and suborbital regions of the left eye, and measurements of horizontal electrooculography were taken between electrodes placed on the outer canthi of the left and right eyes. The impedance of the electrodes was less than 10 kΩ, and homologous sites were within 1 kΩ of each other. Quik-Gel (El Paso, TX, USA) was used as the conducting medium. The signals were amplified using a Neuroscan SynAmps^2^ amplifier unit (El Paso, TX, USA) with a bandpass of 0.05 to 200 Hz, and digitized at a sampling rate of 1000 Hz.

**Figure 1:**
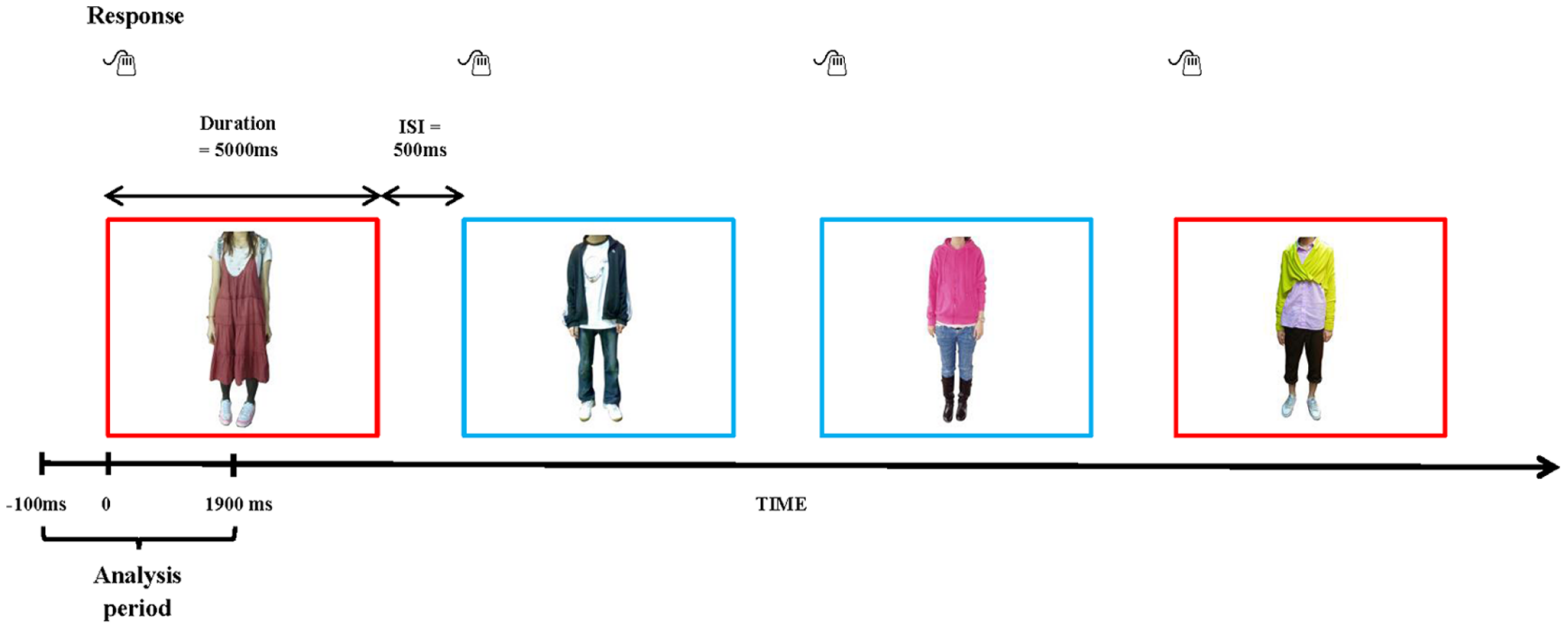
Experimental stimulus presentation procedure. Within each run, 80 images (40 male and 40 female, of which 10 fashion-oriented/beautiful, 10 fashion-oriented/ugly, 10 casual/beautiful and 10 casual/ugly) were presented, randomized by gender (male versus female) and appearance style (fashion-oriented versus casual). Participants made their aesthetic judgments by pressing the buttons on the response pad. Red rectangles indicate examples of fashion-oriented styles judged to be ugly by participants and blue rectangles indicate examples of casual styles judged to be beautiful.

During the EEG recording, participants were invited to make aesthetic judgments on 80 appearance styles represented by 80 pictures of male and female clothing, each visible for 5 seconds with an inter-trial interval of 5.5 seconds. The stimuli were generated and controlled by the stimulus presentation software Neuroscan Stim2 Complete System (El Paso, Texas, USA) using a Dell Optiplex 745 and presented to participants in the middle of a 19-inch color CRT monitor (DiamondDigital DV998FDB). Participants responded to the pictures by pressing the buttons on a STIM Response Pad. The stimuli were randomly presented in terms of gender (male versus female) and appearance styles (fashion-oriented versus casual) (Fig. 1). Among the behavioral measures were the time taken by participants to make aesthetic judgments and the number of personal-appearance styles perceived as beautiful or ugly. After the judgment task, baseline measurements were taken over 5 minutes during which participants were asked to relax fully and look at the blank screens of their respective computer monitors.

### EEG Processing

The EEG data for each participant were imported into the EEGLAB software program using MATLAB 7.9.0. To capture the EEG segments corresponding to styles perceived as beautiful and ugly, the data were epoched separately for each “beautiful” or “ugly” judgment made by each participant in the interval from 100 ms before the stimulus onset to 1900 ms after the stimulus. Next, the segmented epochs for the styles perceived to be beautiful or ugly and the EEG data collected over 5 minutes in the resting state underwent offline processing for artifact removal using the NeuroGuide software program (NeuroGuide, v2.7.9). Using a built-in function of the NeuroGuide software, split-half reliability tests and test-retest reliability tests were conducted on the selected EEG segments. Only data with >90% reliability that were free of artifacts for at least 1 minute in the resting state or contained at least 25 artifact-free epochs for “beautiful” and “ugly” were selected for the subsequent spectral analysis. Fast Fourier transformation was used to translate the signals to the frequency domain. The resting EEG data and the epochs for the styles considered beautiful and ugly were then analyzed over the 64 electrode positions to measure theta coherence [42], [43] and frontal alpha asymmetry [35], [37], [44]–[46].

#### Theta coherence

Theta coherence, defined as the spectral cross-correlation between two signals normalised by their power spectra [47]–[49], was calculated for the control stage (resting with eyes open) and the experimental stage (aesthetic judgments of appearance styles) between all of the electrode pairs, except the eight midline electrodes (Fpz, Fz, FCz, Cz, CPz, Pz, POz, Oz). Fisher's *z*-transform was used to produce a Gaussian distribution from the coherence values. Following the published literature [50]–[52], the values obtained were inverse-transformed for reporting. They ranged from 0 to 1, with higher values representing stronger phase synchronization between the signals of two electrode pairs. “Short-range intrahemispheric coherence” denotes the synchronization between adjacent electrodes, and is a measure of connectivity within the same hemisphere. “Long-range coherence” describes the synchronization between any electrode pairs that are separated by at least one electrode, and reflects the connectivity between two distal regions within or between hemispheres.

Within each hemisphere, the theta-coherence values for possible electrode pairs were further averaged and categorized as follows: (i) short-range intrahemispheric coherence (between adjacent electrode pairs, such as F1-F3, C3-C5, P5-P7 (left) versus F2-F4, C4-C6, P6-P8 (right)); or (ii) intrahemispheric long-range coherence (separated by at least one electrode, such as F1–C1, C3-P3, F5-P5 (left) versus F2-C2, C4-P4, F6-P6 (right)). Intra-hemispheric frontoposterior coherence values were obtained for electrode pairs such as F3-P3 and F8-O2. Interhemispheric coherence was calculated separately within the following cortical regions: (i) frontal (Fp1–Fp2, AF3–AF4, F1–F2, F3–F4, F5–F6, F7–F8), (ii) central (FC1–FC2, FC3–FC4, FC5–FC6, C1–C2, C3–C4, C5–C6, CP1–CP2, CP3–CP4, CP5–CP6), (iii) temporal (FT7–FT8, T3–T4, TP7–TP8), and (iv) parietal/occipital (P1–P2, P3–P4, P5–P6, P7–P8, PO3–PO4, PO5–PO6, PO7–PO8, O1–O2) [42], [43], [52].

#### Frontal Alpha Asymmetry

The most commonly used measure of the level and direction of asymmetry is the alpha-asymmetry index, derived by subtracting the log-transformed absolute alpha power of the left hemisphere from the analogous log-transformed alpha power of the right hemisphere (i.e. log right – log left) [32]. This is a well-documented method of computing alpha asymmetry in the prefrontal region, and is based on a formula proposed by Davidson [53], who conducted a series of studies on the use of this asymmetry index to reflect human mood states, and repeatedly identified a positive association between left-sided activation and positive mood.

On the basis of Davidson's findings [27]–[29], [31], [32], [53], which link frontal asymmetric activation with positive emotion, the asymmetry index was used to examine frontal asymmetric activation at the anterior electrode sites of F5/F6 during the control stage (resting with eyes open) and the experimental stage (aesthetic judgments of appearance styles). As alpha power is inversely associated with activation, a positive asymmetry score denoting greater alpha activity on the right and less alpha power on the left suggests greater left-sided activation, that is, a more positive emotional response. Conversely, a negative score represents greater activation on the right side, suggesting a more negative emotional response.

### Statistical Data Analysis

The statistical data analysis was carried out using SPSS Version 21.0 for Windows (SPSS Inc, Chicago IL, USA). Kolmogoroff-Smirnov tests were used to confirm the normal distribution of the data. As all of the variables exhibited a normal distribution, parametric statistics were used for comparison. Repeated-measures analysis of variance (ANOVA) with three within-subject factors—Condition (beautiful, ugly and resting), Hemisphere (left and right) and Range (short and long)—was used to compare intrahemispheric theta coherence. Interhemispheric coherence was compared using repeated-measures ANOVA with two within-subject factors: Condition (beautiful, ugly and resting) and Region (frontal, temporal, central and parietal/occipital). Repeated-measures ANOVA with one within-subject factor, Condition (beautiful, ugly and resting), was also performed to measure frontal alpha asymmetry. Partial eta-squared (partial η^2^) values were calculated as effect size indices for all of the ANOVA tests. Post-hoc comparisons between the three conditions were made using paired-sample *t*-tests with 14 degrees of freedom. A significance level of *p*<0.01 was used as a partial correction for the multiple comparisons, but results with significance levels of *p*<0.05 are also reported to show the overall trends.

## Results

### Behavioral performance

Of the 80 personal-appearance styles, 97.5% were rated by participants as either beautiful or ugly; no responses were made to 3.5% of the styles. Table 1 provides the mean response times (computed only for the trials with responses), the number of appearance styles perceived as beautiful and ugly, and their standard deviations. Although longer latency periods were observed for aesthetic judgment of appearance styles perceived to be beautiful, neither the difference in response time nor the difference in the number of styles judged were significant (*p*>0.05).

**Table 1:** Mean judgment latency and number of personal-appearance styles perceived as beautiful or ugly during the EEG experiment.

	Beautiful	Ugly	*t*-test	*p*-value
Responses time (ms)	1932.34±636.92	1679.18±415.17	1.993	0.066
No. of styles being perceived	36.60±6.03	39.53±5.18	−1.178	0.259

### Theta coherence

**Figure 2:**
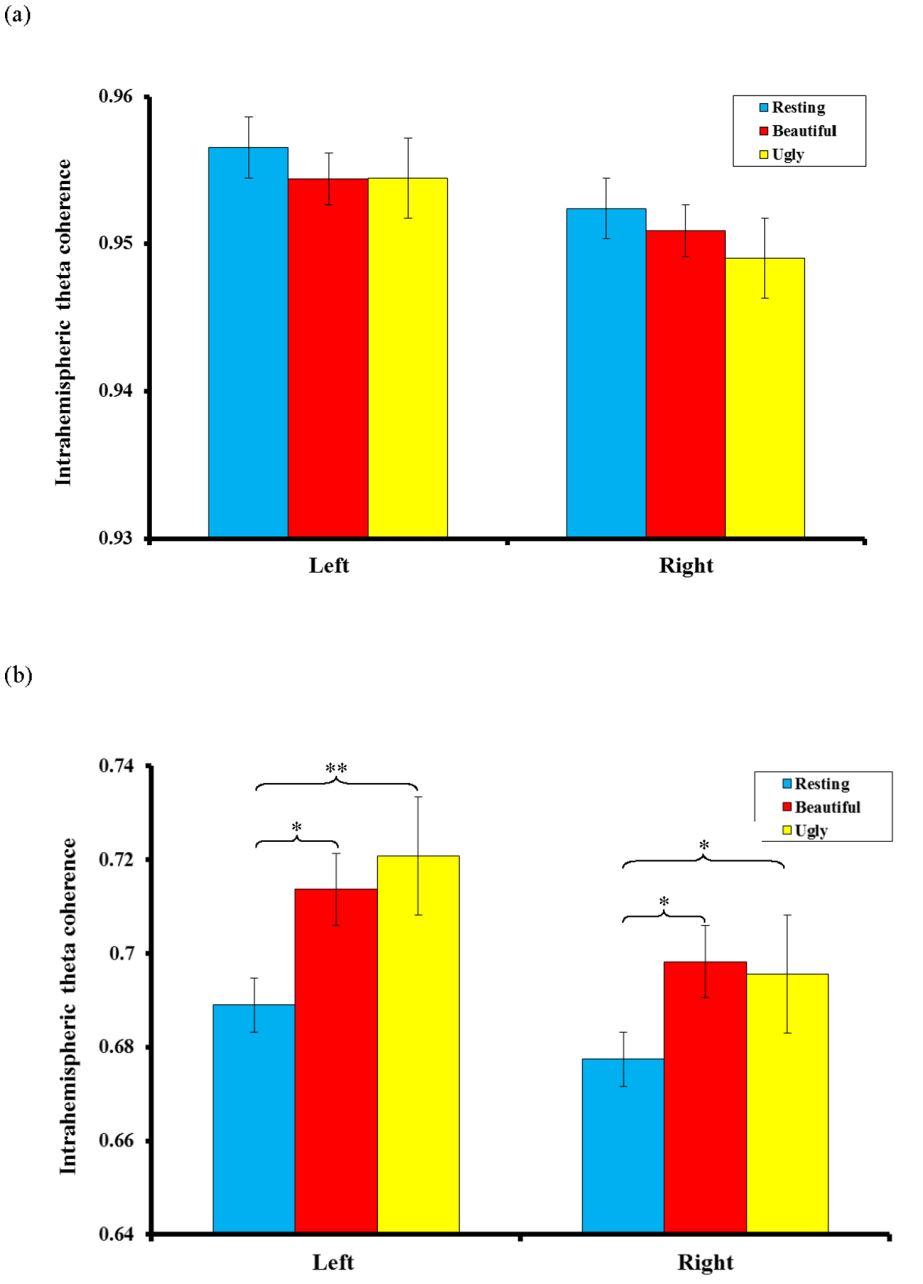
Mean (inverse Fisher's z) values in the theta (4–8 Hz) frequency band for intrahemispheric short-range coherence, (a), and long-range coherence, (b). Compared with the resting measurements, long-range theta coherence increased in both the left hemisphere and the right hemisphere when the personal-appearance styles were judged to be beautiful or ugly (* *p*<0.05; ** *p*<0.01).

Repeated-measures ANOVA was conducted for Condition x Hemisphere x Range to compare intrahemispheric coherence in the theta-frequency band. The results showed that the interaction effect of Condition and Range was significant (*F*(2, 13) = 9.327, *p* = 0.003, partial η^2^ = 0.589). Subsequent post-hoc comparisons revealed that compared with the resting measurements, long-range theta coherence increased in both hemispheres when personal-appearance styles were judged to be either beautiful (left: *t* = 2.844, *p* = 0.013; right: *t* = 2.302, *p* = 0.037) or ugly (left: *t* = 3.425, *p* = 0.004; right: *t* = 2.399, *p* = 0.031). The differences in both hemispheres were near to the corrected significance level of *p*<0.01. However, there was no significant difference in long-range theta coherence between judgments of beautiful and ugly, and no significant differences in short-range theta coherence were observed between the three conditions in either hemisphere (Fig. 2).

**Figure 3:**
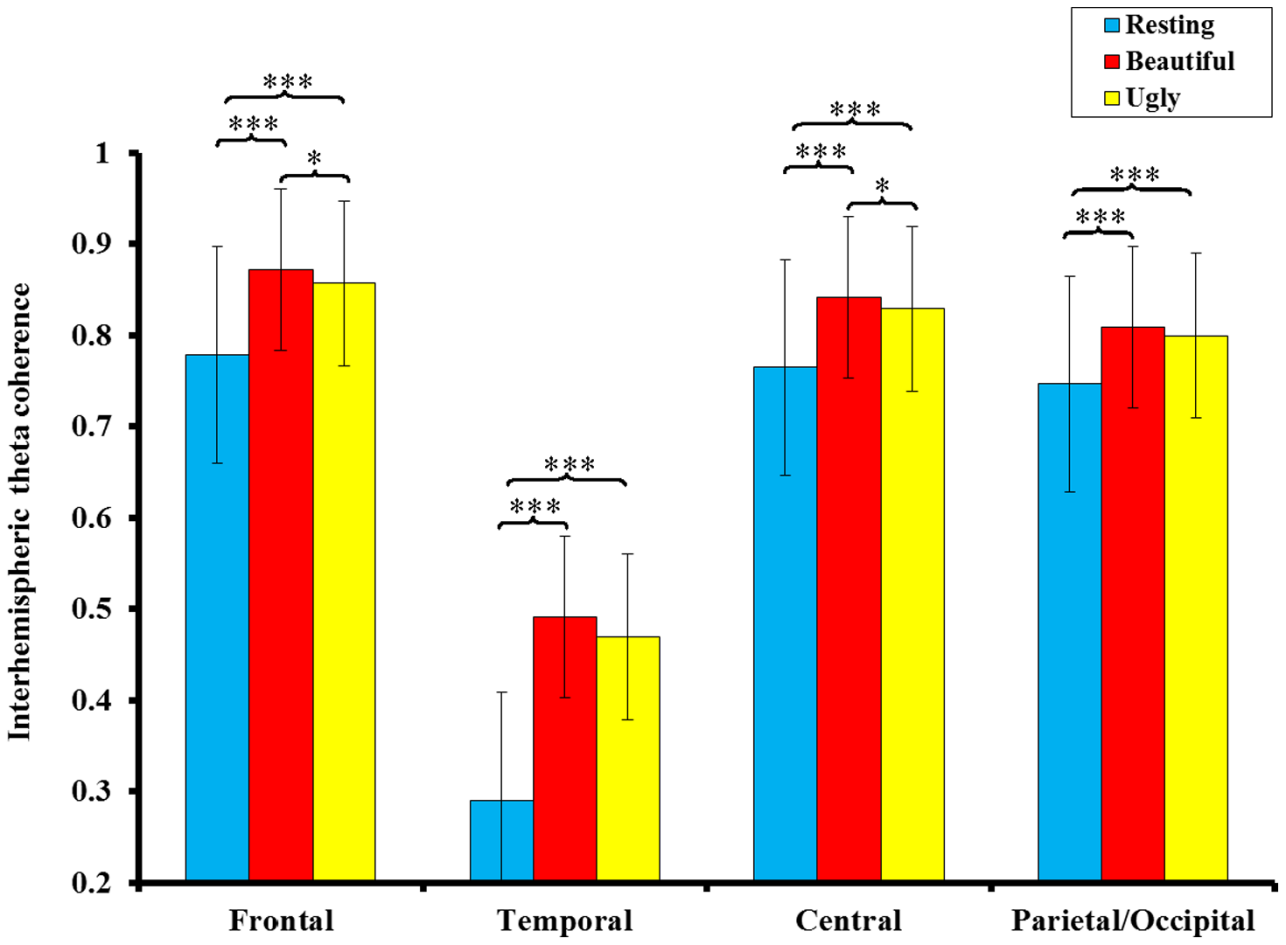
Mean (inverse Fisher's z) values in the theta (4–8 Hz) frequency band for interhemispheric coherence in the frontal, temporal, central and parietal/occipital cortical regions. Compared with the resting measurements, theta coherence increased in the frontal, temporal, central and parietal/occipital cortical regions when the personal-appearance styles were judged to be beautiful or ugly. The judgment of personal-appearance styles as beautiful elicited greater theta coherence in the frontal and central cortical regions, compared with personal-appearance styles deemed to be ugly (* *p*<0.05; *** *p*<0.001).

Repeated-measures ANOVA was used to compare the three conditions in terms of interhemispheric theta coherence in four cortical regions. The results revealed that the main effects of Condition (*F*(2, 13) = 49.425, *p* = 0.000, partial η^2^ = 0.884) and Region (*F*(3, 12) = 195.88, *p* = 0.000, partial η^2^ = 0.980) and the interaction effect of Condition and Region (*F*(6, 9) = 5.052, *p* = 0.016, partial η^2^ = 0.771) were significant, indicating the existence of significant and location-specific differences in interhemispheric theta coherence between the three conditions. Subsequent post-hoc comparisons showed that compared with the resting measurements, interhemispheric theta coherence increased in the frontal, temporal, central and parietal/occipital regions when personal-appearance styles were judged to be either beautiful (frontal: *t* = 9.068, *p* = 0.000; temporal: *t* = 8.093, *p* = 0.000; central: *t* = 6.984, *p* = 0.000; parietal/occipital: *t* = 6.884, *p* = 0.000) or ugly (frontal: *t* = 6.049, *p* = 0.000; temporal: *t* = 7.174, *p* = 0.000; central: *t* = 5.375, *p* = 0.000; parietal/occipital: *t* = 6.640, *p* = 0.000). Compared with judgment of personal-appearance styles as ugly, judgment of styles as beautiful elicited greater interhemispheric theta coherence in the frontal (*t* = 2.674, *p* = 0.018) and central (*t* = 2.122, *p* = 0.05) regions (Fig. 3).

### Topographic map of theta coherence with task difference

**Figure 4:**
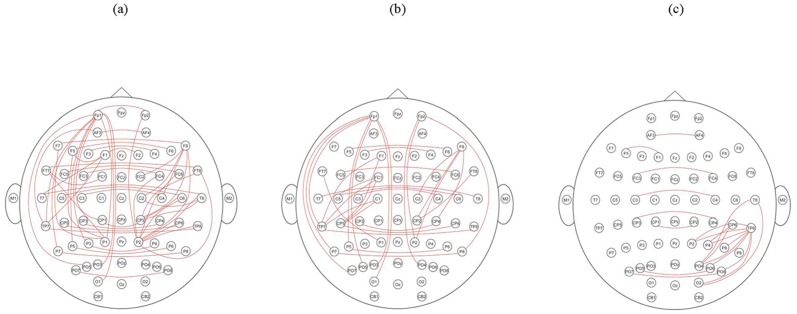
Differences in theta coherence between (a) “beautiful” versus resting; (b) “ugly” versus resting; and (c) “beautiful” versus “ugly.” Paired Wilcoxon tests were used as statistical filters, and the resulting error probabilities (*p*<0.0001) were mapped onto the topographic map as connecting lines between the electrodes involved.

Paired Wilcoxon tests were used to obtain an overall topographic pattern of the differences in coherence between the three conditions. Normally, significance levels are adjusted to prevent the inflation of error probability. However, given the large number of coherence values obtained for several conditions from multiple comparisons, such an adjustment would have resulted in an extremely low probability of rejecting a false null hypothesis. Therefore, the error probabilities for *p*<0.0001 were chosen and plotted on the topographic maps. The connecting lines between corresponding electrodes provided clues to the possible differences in coherence between conditions (Fig. 4).

Although the post-hoc comparisons indicated that the difference was near to the corrected significance level of *p*<0.01, the interhemispheric theta coherence increased at some electrodes sites in the frontal and central regions when personal-appearance styles were judged to be beautiful rather than ugly. In addition, beautiful personal-appearance styles also elicited greater long-range intrahemispheric theta coherence than ugly personal-appearance styles in a small number of posterior electrode sites in the right hemisphere (Fig. 4c).

### Frontal Alpha Asymmetry

**Figure 5:**
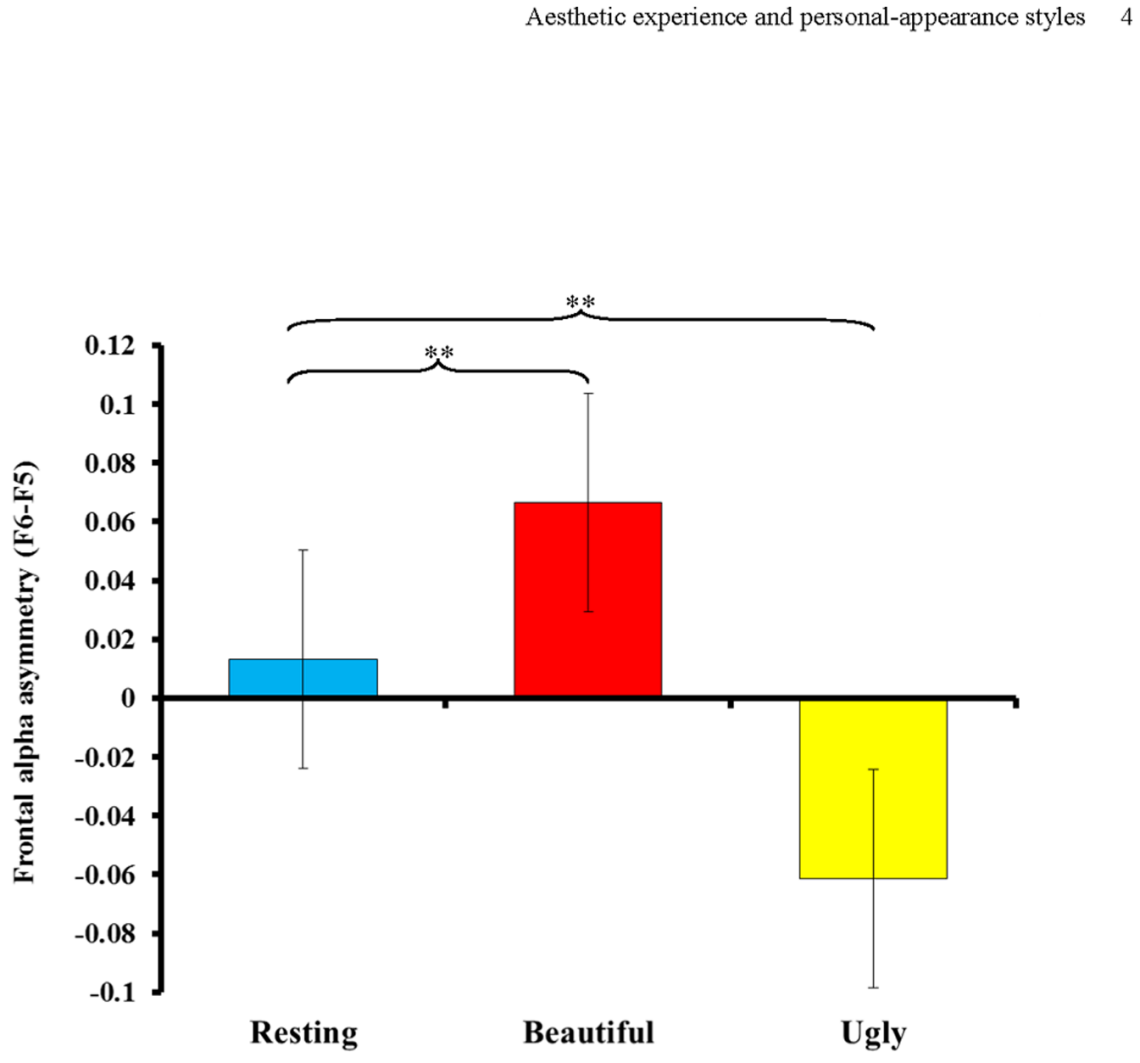
Frontal alpha asymmetry in response to personal-appearance styles perceived as beautiful or ugly and in the resting state. As compared with the resting measurements, positive frontal alpha asymmetry was observed when participants deemed personal-appearance styles beautiful, and negative frontal alpha asymmetry was exhibited when the personal-appearance styles was judged to be ugly (** *p*<0.01).

Repeated-measures ANOVA was conducted to compare the frontal alpha asymmetry values obtained for the three conditions. The results revealed that the main effect of Condition was significant (*F*(2,13) = 8.512, *p* = 0.004, partial η^2^ = 0.567), indicating significant differences in frontal alpha asymmetry between the three conditions. Subsequent post-hoc comparisons revealed that compared with the resting EEG measurements, more positive frontal alpha asymmetry (*t* = 3.794, *p* = 0.002) was found when personal-appearance styles were viewed as beautiful, and more negative frontal alpha asymmetry was observed when personal-appearance styles were judged to be ugly (*t* = −3.205, *p* = 0.006) (Fig. 5).

## Discussion

According to Chatterjee's model [2], the visual attributes of an aesthetic object, like those of any other visual object, are processed in the occipital brain regions. The attributes of an aesthetic object engage attention, and attention in turn enhances the processing of the attributes. Attentional processing in the fronto-parietal circuits continuously modulates the neural processing of visual attributes within the ventral visual system [3], [4]. With regard to attentional processing, Jacobsen et al. [24] found that aesthetic judgments activated not only the frontomedian cortex but the left intraparietal sulcus of the symmetry network. In contrast, Vartanian and Goel [17] reported increased activation in the occipital cortex in response to an increasing preference for a visual stimulus, suggesting that the visual attributes of preferred stimuli receive enhanced processing. However, it is difficult to determine whether increased occipital activity is associated with the attentional or emotional modulation of visual processing, as Lane et al. [54] found emotional valence, arousal and attention to have common effects on the neural activation of visual processing. In the current study, the quantitative EEG measure of theta coherence was used to further explore whether localized and/or interregional theta coherence is engaged during aesthetic judgments of personal-appearance styles. Consistent with our hypothesis, aesthetic judgments of personal-appearance styles resulted in elevated theta coherence, as compared with the resting measurements. This increase was only observed in the long-range frontal-parietal regions of the left and right hemispheres during the process of aesthetic judgments, and there was no significant difference in localized short-range theta coherence between the control stage (resting with eyes open) and the experimental stage (aesthetic judgments of appearance styles). As localized theta coherence is more closely related to the neural correlates of an attention system, the insignificant difference between the control and experimental conditions implies that the allocation of attentional load was similar during the control and experimental stages. The volume conduction of current through the tissues of the head can raise the EEG coherence at moderately separated (<10 cm) electrodes. Lower frequency EEG coherence is thought to result from a mixture of volume conduction effects and genuine source coherence [55]. It is thus also conceivable that the absence of a significant difference in the localized short-range theta coherence between the control and experimental stages was the result of the volume conduction problem. However, the increase in long-range frontal-parietal theta coherence during aesthetic judgments suggests that central executive processing is related to aesthetic judgments. Previous studies have indicated that long-range coherence in the theta-frequency range is related to various cognitive processes, such as working-memory retention [56], the top-down processing of mental images [57], the performance of mental tasks requiring focused attention and working-memory resources [58], [59], and mental-calculation tasks [23]. Recent studies have also indicated that interregional coherence, particularly between the prefrontal and parietal brain regions and in the theta-frequency range, is associated with integrative processes mediated by a central executive system. These processes integrate various memory- and information-processing functions [20], [22] and provide the critical neural correlates underlying decision making during goal-directed behavior [60]. However, Baddeley [61] has reported that a central executive system largely mediated by the prefrontal cortex is responsible for various working-memory functions, and provides an interface between memory systems by coordinating encoding and retrieval processes. Therefore, the increase observed in the current study in long-range fronto-parietal theta coherence in the left and right hemispheres during aesthetic judgments offers further support for Chatterjee's model of the integrative processes involved in attention [2], and emphasizes the contribution of these processes to the aesthetic evaluation of personal-appearance styles. Our findings suggest that when making aesthetic judgments of personal-appearance styles, an individual integrates the visual attributes of these styles, such as the design elements of color, pattern and rhythm, and principles such as balance, unity and harmony, with a unique mental aesthetic algorithm used to identify and understand aesthetic patterns [62]. This choice-relevant information is then synthesized to elicit a decision regarding aesthetic preference.

Compared with personal-appearance styles perceived to be ugly, personal-appearance styles judged to be beautiful were found to produce greater frontal and central interhemispheric theta coherence. This increase in interhemispheric coherence indicates the involvement of certain networks in these regions that transfer information between hemispheres, in addition to cortico-cortical neural networks responsible for conveying information within hemispheres. Such an increase usually occurs when more demanding tasks are undertaken and more efficient information processing is required [63]. Therefore, the results imply that the evaluation of an appearance style as beautiful requires more integrative neural processing than the evaluation of a style as ugly. This may also be reflected in the slightly longer response time taken to assess styles as beautiful, although the difference in response time between “beautiful” and “ugly” judgments was not significant. In fact, an early frontocentral negativity effect (400 to 480 ms) has been consistently observed during the aesthetic evaluation of “not-beautiful” patterns, suggesting an early impression formation. This effect has not been observed during the aesthetic evaluation of beautiful patterns [13], [64], [65]. Therefore, it seems that individuals form impressions and make judgments of ugly objects more rapidly than they assess beautiful objects, but that the aesthetic judgments of beautiful objects involves additional neural networks to integrate the relevant information, resulting in longer decision times. A recent event-related potential (ERP) study supported this speculation and found a larger positivity in the peak amplitudes of the early P1 and late P3b components in the posterior and anterior brain regions, respectively, in response to attractive faces than unattractive faces. The differences in the timing and location of these components implied the involvement of multiple processes in judging attractive faces. The later P3d effect observed at 330 to 500 ms after stimuli onset demonstrated that more cognitive resources were required to detect attractive faces [66]. The widely distributed brain regions involved in aesthetic judgments that have been identified in the neuroimaging studies overlaps the functionally connected brain networks responsible for reward representation, affective motor planning, attention-related sensory processing, and evaluative judgments on social and moral cues [67]. These regions include the medial orbitofrontal cortex [15], [16], [68]–[70], the frontomedian cortex [24], [71], the prefrontal cortex [24], the posterior cingulate gyrus [24], [71], the left temporal pole [24], the temporoparietal junction [24], the left angular gyrus [70], the left superior temporal gyrus [70], and the amygdala [71]. Additional brain regions are recruited and brain activity is greater in the orbitofrontal cortex [24], [70], [72], the right amygdala [73] and the secondary visual cortex [71] for stimuli judged to be beautiful than for stimuli judged to be ugly. In addition, a delayed dynamics brain network is more synchronized during aesthetic judgment of beautiful stimuli, compared with the not beautiful one. This delayed aesthetic network consists of the medial occipital, the lateral occipital, the lateral posterior parietal, the medial parietal, the medial frontal and the prefrontal regions in the left hemisphere [69]. Therefore, our findings are consistent with these studies that aesthetic judgment of beautiful objects engages a delayed synchronized brain network covering distinct and widespread brain regions.

Another interesting finding of the current study concerns aesthetic emotion, which is modeled by both Leder et al. [1] and Chatterjee [2] as another output of aesthetic appreciation. The role of emotion during aesthetic appreciation has been investigated from a behavioral perspective [74], [75]. Several fMRI studies have revealed that aesthetic appreciation correlates with activation in several brain regions related to emotion [76], such as the temporal pole [24], the bilateral insular cortex [77], the orbitofrontal cortex [14], [15], the caudate nucleus and the anterior cingulate cortex [17]. These studies have shown that positive aesthetic experiences involve affective processes related to the reward value of the aesthetically judged stimuli. A recent study showed that functional activity in the right amygdala increased when images were judged to be beautiful, compared with assessments of images as ugly [73]. However, it is impossible to determine with certainty from these studies whether a pleasurable emotional experience is elicited during aesthetic judgments. The results of numerous EEG studies of affective processes have suggested that different emotions are associated with different EEG patterns in the frontal regions of the brain [25], [26]. Specifically, frontal alpha asymmetry has been shown to change in response to affective-state manipulation. Researchers have consistently found that environmental stimuli eliciting positive or approach-related emotions (such as joy) produce greater left frontal activation than environmental stimuli encouraging negative or withdrawal-related emotions (such as disgust, fear and sadness). The latter emotions produce greater right frontal activation [25], [78], [79]. Among the environmental stimuli to have received attention are facial expression [78]–[80], emotion-provoking films [31], [81], taste [82], [83] and odor [84]. In the current study, frontal alpha asymmetry was used as an index of human mood states to further explore whether the aesthetic judgment of personal-appearance styles as beautiful results in a pleasurable experience for the perceiver. Compared with the resting measurements, positive frontal alpha asymmetry (suggesting a positive emotional state) was observed when participants judged personal-appearance styles as beautiful, and negative frontal alpha asymmetry was exhibited when personal-appearance styles were judged to be ugly, suggesting a negative emotional state. Therefore, positive affective responses or approach-related emotions were elicited when the individuals viewed personal-appearance styles perceived as beautiful, whereas negative affective responses or withdrawal-related emotions were elicited by personal-appearance styles perceived to be ugly. These findings are consistent with our hypothesis and have significant implications for fashion design or styling, visual merchandising and consumer behavior. If a personal-appearance style worn by an individual or displayed in a store is perceived as beautiful, the perceiver will experience a more pleasant or approach-related emotional response. Although the current study did not investigate participants' behavior following the observed state change, the efficacy of mood change as a predictor of subsequent behavior has been explored in a series of studies by Harmon-Jones and colleagues [85], [86]. The results of these studies suggest that emotional responses are likely to elicit corresponding behavior; that is, “coping” or “motivated” (approach or withdrawal) behavior. This may explain why appearance styles designed for window displays or as fashion highlights in stores can substantially affect consumer behavior, such as increasing the number of store visits or encouraging product purchase. If such products are perceived as beautiful by a consumer, they may trigger a positive or approach-related emotional response that motivates the consumer's subsequent behavior. In addition, our findings highlight the significance of aesthetically pleasing fashion items. Fashion design is a form of art devoted to the creation of clothing, accessories and even lifestyle, and its aim is to satisfy consumers' desire for functional and/or aesthetically pleasing items [87]. The emphasis on aesthetic styling in fashion design is not only a means of product differentiation [88]; it also arises from the insight that fashion design with aesthetic value seems to trigger certain positive responses in consumers, or fulfill their hedonic needs. As a result, consumers are eventually more willing to pay for such items [89], or develop a stronger desire to possess them [90]. Therefore, brain activity in response to personal and “runway” appearance styles could be compared to determine whether consumers' experience of aesthetically designed fashion stimulates different behavioral and neural mechanisms. Runway appearance styles are expected to trigger more prominent positive or approach-related emotional responses than appearance styles displayed by individuals.

One limitation of the present study arises from its biased gender distribution (12 of the 15 participants were female). Recent studies have demonstrated gender differences in the neural underpinnings of aesthetic appreciation, in terms of decision latency and hemispheric lateralization. In response to attractive faces, men have been found to show a slower response, make later decisions and attribute more value to facial appearances than women [66]. A study using repetitive transcranial magnetic stimulation showed that women exhibited stronger right hemisphere dominance than men when appreciating the beauty of the body [91]. Cela-Conde et al. [92] also explored the differences between the neural correlates of aesthetic appreciation in men and women, and found the sexes to exhibit significantly different activity in the parietal region when they judged a stimulus as beautiful. The male individuals' parietal activity was lateralized to the right hemisphere, while the female individuals exhibited more bilateral parietal activity. This gender-related difference in the neural correlates of aesthetic judgments may be due to differences between males and females in their strategies for spatially processing beauty. As the results of this study show, aesthetic judgments are associated with integrative processes that also involve the frontal and parietal cortical regions. Therefore, men may exhibit more lateralized elevated long-range fronto-parietal theta coherence in the right hemisphere, and women may demonstrate increased long-range fronto-parietal theta coherence bilaterally in both hemispheres. In this study, the stimuli were categorized into four major groups, namely male fashion-oriented styles, male casual styles, female fashion-oriented styles and female casual styles. EEG data were extracted into epochs according to gender (male versus female) and appearance style (fashion-oriented versus casual) categories to investigate the differences in brain activity between these categories. However, some of the categories did not contain enough artifact-free epochs to ensure a reliable EEG data analysis. Nevertheless, it would be interesting to determine whether individuals of different genders exhibit differences in brain activity in response to appearance styles worn by men or woman, and thereby gain insight into the influence of sex preferences on the aesthetic judgments of appearance styles. This subject has rarely been explored in relation to other environmental stimuli, such as painting or musical excerpts. Do individuals experience more positive or approach-related emotional responses to appearance styles worn by the opposite sex and judged to be beautiful? The findings of this research would shed further light on the effects of social interaction on aesthetic judgments.

Individual personal perceptual style (local versus global) has also been found to be a crucial factor that determines the degree of pleasure experienced when viewing artworks. Individuals with a local perceptual style have been shown to pay more attention and exhibit a more pleasurable experience during the aesthetic appreciation of ambiguous artworks. No significant differences have been found between individuals with local and global perceptual styles when viewing unambiguous artworks [93]. It would thus be worthwhile to explore how brain activity differs in the central processing and emotional responses of individuals with local or global perceptual styles during the aesthetic judgments of appearance styles, as the appearance styles created by consumers convey symbolic ambiguity [94]. Finally, Cupchik and Laszlo [95] identified two distinct forms of art appreciation: pleasure-based and cognitively based. Experts in an environmental stimulus such as art have knowledge that supports cognitive processing, so their perception of the stimulus is more cognitively based. Individuals with less expertise in the given stimulus engage in a more emotional form of art appreciation [96]. Kirk et al. [97] provided initial explanatory evidence of the effects of expertise in architecture on the cognitive processing and affective responses involved in aesthetic judgments. It is thus assumed that expertise in fashion modulates the neural processing of individuals' aesthetic judgments of appearance styles. The brain activity associated with central processing and emotional responses during aesthetic judgments also differs between experts—such as fashion designers, stylists and visual merchandizers—and amateurs, which deserves further investigation.
